# A scoping review of the factors affecting work–life balance among nurse educators

**DOI:** 10.4102/hsag.v30i0.2910

**Published:** 2025-07-24

**Authors:** Letta Mathebula, Rirhandzu F. Mathevula, Tshiamo N. Ramalepa

**Affiliations:** 1 Department of Nursing Sciences, School of Healthcare Sciences, Sefako Makgatho Health Sciences University, Pretoria, South Africa

**Keywords:** work–life balance, nurse educators, academics, factors, nursing educational institution, scoping review

## Abstract

**Background:**

There is a dearth of research on the factors that contribute to a lack of work–life balance (WLB) among nurse educators because most studies focused on nurses or academics in general rather than nurse educators. Thus, more studies are needed to explore the factors that contribute to the lack of WLB among nurse educators.

**Aim:**

To explore and investigate the scope and quantity of literature on the factors affecting the WLB among nurse educators.

**Method:**

This scoping review followed Arksey and O’Malley’s framework, using the Joanna Briggs Institute (JBI) Manual for Evidence Synthesis to extract and chart sources. Seventeen articles (2014–2024) were analyzed using descriptive analysis, PRISMA-ScR, and thematic analysis.

**Results:**

The scoping review identifies factors and strategies impacting WLB among nurse educators, highlighting occupational stress, understaffing, and heavy workloads as key contributors to imbalance. It also explores the role of technology in improving WLB.

**Conclusion:**

The scoping review identified factors that contribute to work–life imbalance among nurse educators and proposed strategies to address these issues. Studies suggest that WLB programmes can also lower employee stress levels. Furthermore, the review highlights a significant gap in the literature concerning the factors contributing to the lack of WLB among nurse educators in the sub-Saharan region.

**Contribution:**

The findings of this scoping review might assist in the development of strategies that could help nurse educators deal with the factors that contribute to the lack of WLB among nurse educators in nursing education institutions.

## Introduction

Work–life balance (WLB) is a state of equilibrium between the demands of work with the individual’s personal life and the family, thus decreasing the possibility of conflicts in work and personal life (Fadillah, Aras & Wahyuni 2022). The subject of WLB is becoming more prevalent among employees and management in many institutions due to various societal, economic and cultural shifts that have reshaped priorities in the workplace. This shift reflects a broader understanding that well-being and productivity are interconnected and that a supportive work environment can lead to higher job satisfaction, reduced stress levels and overall improved performance. This includes institutions such as universities and hospitals where other categories of health professionals are exposed to strenuous long working hours and other factors such as a lack of administrative support, inadequate preparation, increased workload and stress and role uncertainty (Laari et al. 2024). Hence, achieving a healthy WLB is a significant issue in the field of nursing education (Xu & Zhao 2024). In recent years, there has been a growing recognition of the impact of WLB on employees and their families (Tamunomiebi & Oyibo 2020). The professional responsibilities and demands placed upon nurse educators frequently pose challenges to their WLB. To succeed in teaching, educators need to find a balance between their personal and professional lives (Jimenez & Viloria 2024). Moreover, when individuals place equal importance on their personal and professional commitments, they achieve a WLB (Ganapathi, Aithal & Kanchana 2023).

Shabir and Gani (2020) reflected that work and family issues are increasingly relevant for institutions to consider as the structure and makeup of the employees continue to change. However, the difficulty for any institution lies in maximising individual performance (Tamunomiebi & Oyibo 2020). Moreover, the favourable aspects of their employment encompass social support, intellectually stimulating tasks, equitable remuneration, and provisions for the preservation of the employees. The demanding responsibilities of nurse educators often hinder their WLB, as extended hours and dedication can blur the line between work and personal time. Nurse educators may have increased workload as a result of understaffing, the demands associated with their role, and professional development, which can make it challenging for them to properly achieve their responsibilities and achieve an acceptable WLB.

**FIGURE 1:**
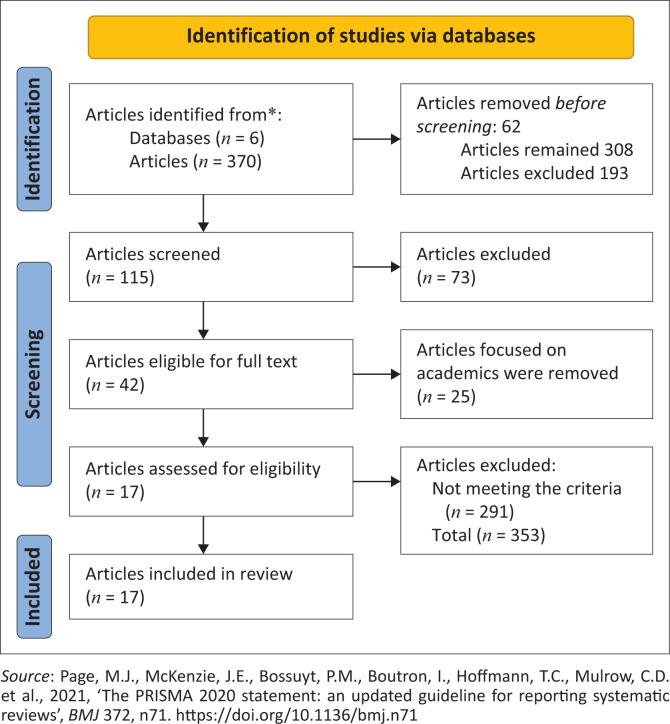
Flow diagram of the scoping review process based on the Preferred Reporting Items for Systematic Review and Meta-analysis.

Understanding the importance of WLB improves physical and mental health and productivity, especially for individuals who are working remotely (Como, Hambley & Domene 2021). In addition to teaching nurses, this position involves addressing the psychological and emotional needs of students. WLB poses several challenges for nurse educators, who often perceive their limitations as personal rather than societal issues (Erasmus, Downing & Ntshingila 2024). Moreover, this emotionally charged working environment can be demanding and detrimental to one’s well-being. It can be challenging for nurse educators to maintain high standards while also assisting students in overcoming difficulties in their academic and professional endeavours. Achieving a good WLB is extremely important for individual performance and institutional achievement (Tamunomiebi & Oyibo 2020); however, high levels of work-related stress and discontent can significantly affect an individual’s personal life by hindering their ability to disengage from work and relax during social time. WLB can be difficult to achieve, and individuals who struggle with this balance may face emotional challenges that can negatively impact their health and pose a significant risk to the global economy (Deepa et al. 2023; Khateeb 2023). Stress and an unbalanced work–life relationship can result from insufficient support from colleagues, managers or institutions. The study aimed to explore and investigate the scope and quantity of the literature on the factors affecting the WLB among nurse educators at Nursing Education Institutions using a scoping review.

## Objectives of the study

- To explore the factors affecting the WLB of nurse educators.
- To investigate the scope and quantity of literature on the factors affecting the WLB among nurse educators.

### Review questions

- *What are the factors contributing to the lack of work–life balance among nurse educators?*
- *What is the scope and quantity of literature available on the factors contributing to the lack of work–life balance among nurse educators?*

## Methods

The review employed a scoping review methodology, which gathered data from diverse sources and synthesised the information logically and reliably. Similarly, literature reviews gather materials from multiple sources, analysing them comprehensively to ensure the accuracy of the information (Peterson et al. 2017). This scoping review followed the methodological framework established by Arksey and O’Malley (Levac, Colquhoun, & O’Brien 2010) and adhered to the updated methodological guidance outlined in the Joanna Briggs Institute (JBI) Manual for Evidence Synthesis by Peters et al. (2020). The process involved a systematic approach, including defining review questions and objectives, setting eligibility criteria, developing a search strategy, screening and selecting evidence, extracting and organising data, analysing the evidence, and presenting the results along with a summary, in alignment with the review’s purpose.

### Eligibility criteria

To provide more accurate and useful data, inclusion and exclusion criteria were developed for selecting the articles from the databases. The measures for the inclusion and exclusion criteria were subsequently implemented throughout all search engines and databases. Studies that met the inclusion criteria included studies that were published in English, full-text articles, peer-reviewed articles, systematic reviews, official reports, studies that were published between 2014 and 2024, dissertations and guidelines and policies. Studies that were excluded include editorials, magazines and books focusing on nursing.

### Search strategy

Searching databases for articles with details on the factors affecting WLB among nurse educators was part of the literature search. Six databases were used in the search strategy, including EBSCO-host, SAGE Journal Online, Google Scholar, Cumulative Index to Nursing and Allied Healthcare Literature (CINAHL), and PubMed Central. The articles searched on these databases were used to review and analyse the relevant studies. The method for choosing sources of evidence was based on the JBI Manual for Evidence Synthesis and the Preferred Reporting Items for Systematic Reviews and Meta-Analysis for Scoping Reviews (PRISMA-ScR) recommendations. The keyword combinations used for the literature search include, ‘nurse educators’, ‘factors contributing and lack of work–life balance’, work–life balance ‘Nurse Educators and work–life balance’, ‘lecturers and work–life imbalance’. The search was expanded by using more keyword combinations such as academics and WLB due to the dearth of literature on factors contributing to the lack of WLB among nurse educators.

### Evidence screening and selection

Following the search, every citation found was gathered and added to the authors’ library in Google Scholar. The chosen sources for the scoping review were then managed and arranged, and duplicates were removed, by importing the references into the Rayyan program. The researchers assessed the abstracts and titles, comparing them with the researchers’ inclusion standards. All possibly relevant sources were retrieved in full, and their citation data were included. The researcher thoroughly reviewed and assessed all the selected sources to ensure they met the criteria for inclusion. Full-text sources of evidence that did not meet the inclusion criteria were excluded, and the scoping review noted and recorded the rationale for these decisions.

### Charting the data

A logical and descriptive summary of the outcomes is provided by data extraction or data charting, which also shows how well the results match the goals of the scoping review. The literature about the factors affecting WLB among nurse educators was the main topic of the data that were extracted. A data-extraction tool that was modified from the JBI Manual for Evidence Synthesis served as guidance for the data extraction or charting procedure. Table 4 illustrates how the tool was used to extract information from the publications about the scoping review, including authors, publication year, source details, methodology and important conclusions.

### Analysis of evidence

The three processes proposed by Arksey and O’Malley (2005) in their framework were followed for analysing the data. The three steps include an analytic framework through the PRISMA-ScR, descriptive analysis and thematic analysis. The first step was creating an analytical framework that provided a broad assessment of the body of research about the methodologies for the factors that lead to a lack of WLB utilising the PRISMA-ScR flow chart. The second step utilised a descriptive statistical analysis using tables to illustrate the types of research chosen. Table 1, Table 2, and Table 3 give the descriptive data reported on the types of publications of sources, the year of publication, and the country of origin of the research. The third and final step involved employing thematic analysis to identify themes associated with the factors affecting WLB among nurse educators. The themes that emerged throughout the study were explored in detail to portray an explanation of the factors that contribute to a lack of WLB.

**TABLE 1:** Year of publication (*N* = 17).

Year of publication	Number of studies	%
2024	4	23.52
2022	3	17.64
2020	3	17.64
2017	2	11.76
2023	2	11.76
2021	2	11.76
2016	1	5.88

*Source*: Ramalepa, T.N. & Netangaheni, T.R., 2024, ‘A scoping review of strategies for adolescents’ sexual and reproductive health role modelling’, *South African Family Practice* 66(1), 1–9. https://doi.org/10.4102/safp.v66i1.5859

**TABLE 2:** Type of publication of sources (*N* = 17).

Type of study	Number of studies	%
Quantitative	7	35.29
Reviews	5	23.52
Qualitative	2	11.76
Mixed methods	2	11.76
Theoretical framework	1	5.88

*Source*: Ramalepa, T.N. & Netangaheni, T.R., 2024, ‘A scoping review of strategies for adolescents’ sexual and reproductive health role modelling’, *South African Family Practice* 66(1), 1–9. https://doi.org/10.4102/safp.v66i1.5859

**TABLE 3:** Country of origin and studies (*N* = 17).

Country of origin	Number of studies	%
Australia	4	23.53
United States	2	11.76
United Kingdom	2	11.76
Indonesia	2	11.76
West Asia	1	5.88
New Zealand	1	5.88
India	1	5.88
RSA	1	5.88
Ghana	1	5.88
Malawi	1	5.88
Zimbabwe	1	5.88

*Source*: Ramalepa, T.N. & Netangaheni, T.R., 2024, ‘A scoping review of strategies for adolescents’ sexual and reproductive health role modelling’, *South African Family Practice* 66(1), 1–9. https://doi.org/10.4102/safp.v66i1.5859

RSA, Republic of South Africa.

According to the categories presented in Table 2, most of the studies were quantitative, (*n* = 7). This was followed by comprehensive reviews (*n* = 5) that synthesised existing knowledge. In addition, (*n* = 2) qualitative studies provided in-depth insights, with (*n* = 2) mixed methods approaches that combined quantitative and qualitative techniques. Finally, (*n* = 2) theoretical framework presented a foundational perspective on the topic reviewed.

Table 4 summarises a few studies according to the authors, article title, study methodology and factors affecting WLB among nurse educators. Five studies addressed occupational stress as a factor affecting WLB among nurse educators, four publications addressed understaffing, competence, and a heavy workload and four articles addressed the use of technology as a factor affecting WLB. The three papers discussed ideas for addressing the lack of WLB among nurse educators. Strategies that focus on factors that can assist nurse educators in achieving WLB are shown in three studies by Singh et al. (2020), Zheng et al. (2016) and Lee et al. (2022). The studies discovered that preventative strategies that employing organisations should use to reduce the impact of occupational stress. Both the availability and implementation of organisational WLB programmes have been shown to reduce employee stress levels. Effective stress management improves an individual’s self-esteem and performance in a stable at work.

**TABLE 4:** A summary of selected studies.

Authors	Title of the article	Research methods	Results
1. Boamah et al. (2022)	Striking a balance between work and play: The effects of work–life interference and burnout on faculty turnover intentions and career satisfaction	Mixed-methods	The results contribute to the increasing amount of research connecting burnout to staff turnover and discontent, emphasising important factors influencing burnout among nurse educators
2. Dube and Ndofirepi (2024)	Academic staff commitment in the face of a role balance between work and personal life: Can job satisfaction help?	Quantitative	The findings showed that work satisfaction was strongly predicted by WLB. Moreover, it was shown that work satisfaction acted as a mediator in the indirect association among WLB and emotional standardising obligations.
3. Erasmus et al. (2024)	Work–life integration among nurse educators: A meta-synthesis.	Review	Work–life balance presents several difficulties for nurse educators, who frequently see their shortcomings as personal rather than social problems
4. Fuller and Mott-Smith (2017)	Issues influencing success: Comparing the perspectives of nurse educators and diverse nursing students.	Mixed-methods	Competency plays a significant role in the challenges nurse educators face in achieving a good WLB. When employees feel underqualified or lack the necessary skills for their roles, it can lead to increased stress and longer working hours, ultimately disrupting the equilibrium between their work and social lives.
5. Ganapathi et al. (2023)	Impact of WLB and Stress Management on Job Satisfaction among the Working Women in Higher Educational Institutions in Namakkal District	Qualitative	Effective stress management boosts self-esteem and performance, creating stability in a chaotic world. By taking control of stress, an individual empowers himself to face challenges confidently, leading to greater productivity and a more fulfilling life.
6. Hayes et al. (2021)	Perceived stress, work-related burnout, and working from home before and during COVID-19: An examination of workers in the United States.	Quantitative	The increased stress during the pandemic stemmed from health concerns and the challenges of relying on technology for communication. Adapting to virtual interactions often led to feelings of isolation and anxiety, significantly impacting mental well-being during this unprecedented time.
7. Laari et al. (2024)	Determinants of Workload and Stress Among New Nurse Educators in Ghana: A qualitative study.	Qualitative	A new nurse educator’s workload and stress levels were determined by several factors, especially when managing multiple courses, handling overcrowded classes, working extended hours, and fulfilling responsibilities beyond the classroom.
8. Lee et al. (2022)	Occupational stress in university academics in Australia and New Zealand.	Review	Workplace stress negatively impacts both individuals and organisations. It can lead to health issues like anxiety and burnout, affecting employees’ well-being and job performance. Organisations may face decreased productivity, higher absenteeism, and increased turnover.
9. Maharani and Tamara (2024)	The occupational stress and WLB on turnover intentions with job satisfaction as mediating.	Quantitative	The results suggest that there is no straightforward connection between job-related stress and the desire to leave a job, but there is a clear link between supporting a balance of a professional and social life and the desire to leave a job.
10. Phuma-Ngaiyaye, Adejumo and Dartey (2017)	Challenges in neonatal nursing clinical teaching to nurse–midwife technicians in Malawi.	Quantitative	The results suggest that brief duration of employment and insufficient qualifications of nurse educators can significantly impact the quality of nursing education. This combination can lead to challenges in preparing future nurses for the demands of the healthcare field.
11. Salju, Junaidi and Goso (2023)	The effect of digitalisation, work-family conflict, and organisational factors on employee performance during the COVID-19 pandemic	Quantitative	The conclusions emphasise that digital literacy of employees is significantly enhanced by the provision of IT training. This training equips them with essential skills and knowledge, allowing them to use and manage digital tools and resources in their work environment.
12. Shabir and Gani (2020)	Impact of WLB on organisational commitment of women healthcare workers: Structural modelling approach.	Quantitative	Work–life imbalance’s implications and the importance of company guidelines in managing the relationship between job and leisure activities.
13. Shen and Slater (2021)	Occupational Stress, Coping Strategies, Health, and Well-Being among University Academic Staff – An Integrative Review.	Review	This review outlines the essential skills of university educators in promoting their health and well-being, ultimately improving satisfaction and productivity in educational institutions.
14. Shirmohammadi, Au and Beigi (2022)	Remote work and WLB: Lessons learned from the COVID-19 pandemic and suggestions for HRD practitioner	Theoretical framework	The study demonstrates that HR practitioners are crucial in assisting employees in effectively coordinating their expertise and demands by working from home.
15. Singh et al. (2020)	Occupational stress facing nurse academics – A mixed-methods systematic review.	Review	The report recommended preventative steps hiring companies may take to mitigate the impact of workplace stress.
16. Urbina-Garcia (2020)	What do we know about university academics’ mental health? A systematic literature review.	Review	Employee mental illness is a result of the actual academic load. Psychological and physical effects on academics have the potential to significantly lower productivity
17. Zheng et al. (2016)	Impact of individual coping strategies and organisational WLB programmes on Australian employee well-being.	Quantitative	Employee stress levels were found to be reduced by both the availability and use of organisational WLB programmes; but surprisingly, there was no correlation established between WLB and employee health

*Source*: Adapted from Ramalepa, T.N. & Netangaheni, T.R., 2024, ‘A scoping review of strategies for adolescents’ sexual and reproductive health role modelling’, *South African Family Practice* 66(1), 1–9. https://doi.org/10.4102/safp.v66i1.5859

Note: Please refer to the full reference list of this article, available at https://doi.org/10.4102/hsag.v30i0.2910, for more information.

WLB, work–life balance.

### Ethical considerations

Ethical clearance to conduct this study was obtained from the Sefako Makgatho Health Sciences University and Sefako Makgatho Research Ethics Committee (reference no: SMUREC/H/249/2023: PG).

## Review findings

A literature search produced 370 articles from six databases and grey literature sources. After removing 62 duplicates, 308 studies were available for review. Upon reading the abstracts, an additional 193 studies were excluded. A total of 115 relevant studies were selected for the scoping review after carefully applying the predetermined inclusion and exclusion criteria. After further review of the titles and abstracts, 73 entries were eliminated for not meeting the inclusion criteria, resulting in 42 articles that were eligible for full-text reading. Due to the limited research on the factors influencing WLB among nurse educators, 25 articles that focused on academics and nurses, but did not address factors affecting WLB, were also disqualified. The articles discussed general aspects of academics and nursing but did not provide specific insights into the factors. Then, overall search results generated 17 articles for analysis. The documents comprised peer-reviewed studies, full text and with references (*n* = 17), quantitative (*n* = 7), qualitative (*n* = 2), reviews (*n* = 5) and mixed-method articles (*n* = 2) and theoretical framework (*n* = 1). The documents were published from Australia, the United States, the United Kingdom, Indonesia, West Africa, New Zealand, India, Republic of South Africa, Ghana, Malawi and Zimbabwe. Data were analysed using a thematic and narrative approach. The factors contributing to WLB among the nurse educators at the nursing education institution were categorised into four themes, occupational stress among nurse educators, understaffing and heavy workload, the use of technology as a factor affecting WLB, and strategies to help nurse educators achieve WLB.

### Article summary characteristics

Table 1, Table 2, Table 3 and Table 4 display the characteristics of the selected articles focusing on the factors affecting WLB in nurse education. The 17 chosen articles were published between 2016 and 2024. Table 1 presents a detailed overview of the publication years for the selected research papers. The breakdown is as follows: one paper was published in 2016, followed by two papers in 2017. A notable increase in publications occurred in 2020, with three papers released that year. The trend continued in 2021, which saw two more contributions to the field. In 2022, another three papers were published, maintaining the momentum. The most recent years, 2023 and 2024, show a further surge, with two papers in 2023 and a significant four papers projected for publication in 2024. This distribution highlights the evolving landscape of research output over the years. The factors contributing to the lack of WLB were identified from various countries, including Australia (*n* = 4), the United States (*n* = 2), the United Kingdom (*n* = 2), Indonesia (*n* = 2), West Africa (*n* = 1), New Zealand (*n* = 1), India (*n* = 1), Republic of South Africa (*n* = 1), Ghana (*n* = 1), Malawi (*n* = 1) and Zimbabwe (*n* = 1) as seen in Table 3. These results indicate a lack of studies discussing the factors affecting the WLB of nurse educators in the sub-Saharan region.

### Contributing factors to work–life imbalance and strategies to improve work–life balance

The review and analysis of the selected articles produced four themes, namely, occupational stress among nurse educators, understaffing and heavy workload, the use of technology as a factor affecting WLB and strategies to help nurse educators achieve WLB.

#### Theme 1: Occupational stress among nurse educators

The lack of balance between work and life can lead to occupation-related stress, which has been demonstrated to have negative consequences for an educator’s physical and mental health. Despite significant advances in recent years in the knowledge of stress, little is known about how exhaustion affects nursing institution turnover intentions and professional satisfaction (Boamah et al. 2022; Lee et al. 2022). Nurse educators face challenges in maintaining a WLB, and studies have shown that they experience stress due to their work duties. They need to possess knowledge, skills, and judgment at all levels of teaching, from individual students to the broader healthcare organisation (O’Neill 2016). Occupational stress refers to the unpleasant psychological and physical repercussions that a person experiences as a result of their job responsibilities, environment or other workplace stressors. According to Hayes et al. (2021), nurse educators experienced stress at work, which was influenced by workload and workplace culture. Working remotely was strange for them; however, it was attributed to coronavirus disease 2019 (COVID-19). Singh et al. (2020) concurred that WLB, workload, resources and flexibility are some of the reasons why nursing educators face occupational stress, including burnout. Another factor contributing to a lack of WLB among nurse educators was the short duration and diversity of clinical placements, a lack of emphasis on clinical education, and clinical staff incompetence (Phuma-Ngaiyaye et al. 2017). When students are assigned for short periods, nurse educators become stressed because they do not have sufficient time to guide them. Studies have shown that the university environment can have a negative impact on students’ physical and emotional well-being (Urbina-Garcia 2020). Furthermore, research also suggests that a range of challenges faced by nursing students can hinder their progress through nursing programmes. Finally, these challenges will lead to stress and burnout, impacting their psychological state.

#### Theme 2: Understaffing and heavy workload

Understaffing in the nursing field happens when there are not enough nurse educators to carry out their responsibilities and do not have the necessary qualifications at nursing facilities in several contexts. Understaffing significantly impacts the WLB of nurse educators by creating unsustainable workloads, such as managing excessively large class sizes. A study by Laari et al. (2024) highlights contributing factors, including teaching multiple classes and courses with large student groups. If the institution has enough staff, nurse educators will not be subjected to large classes. The study conducted by Urbina-Garcia (2020) discovered that there is strong evidence that the pressures placed on the higher education industry globally today are having a negative effect on the mental health of academics nationwide. Employee mental illness is a result of the actual academic load. A heavy workload occurs when the number of responsibilities one has exceeds the limits of what can reasonably be accomplished in a given capacity. Academics’ mental health is influenced by various factors, including teaching, supervision and research funding. In addition, it has also been found that nurse educators may struggle to meet the needs of these students (Fuller & Mott-Smith 2017).

#### Theme 3: The use of technology as a factor that affects work-life balance

Education is a critical human need since it is responsible for generating human resources for national and state development (Soelton 2023). In a study by Schaffer et al. (2016), it was noted that advancements in technology have created opportunities for nurse educators to find new ways of delivering nursing education content. There is also a call from researchers in the nursing field for nurse educators to provide students with real-world experiences that will help them navigate the social complexity within the healthcare organisation (McKinley Yoder & Pesch 2020).

According to Hayes et al. (2021), the key challenges reported were communication, collaboration and time management with employees using technology. Working remotely may lead to increased stress and burnout, raising concerns about some employers’ decision to make it permanent. However, the study done by Kotcherlakota, Kupzyk and Rejda (2017) highlighted that less experienced educators had an increased likelihood of being motivated and having positive attitudes toward the adoption and utilisation of modern technology. Lastly, the study conducted by Salju et al. (2023), indicates that employees’ digital literacy is significantly influenced by information technology (IT) training.

#### Theme 4: Strategies to help nurse educators achieve work–life balance

Institutions and human resource departments worldwide have embraced the concept of WLB. This has led to the development of numerous policies and strategies to address the conflict between work and personal life (Khateeb 2021). In addition, higher learning institutions create outstanding personnel and are supported by excellent competency in educators. Educators must be reliable and appear professional to give shape to succeeding numerous generations that are skilled and have qualities (Soelton 2023). Nurse educators experience less work-related stress when they have a better WLB (Fadillah et al. 2021). Based on the evidence of the reviewed articles the following recommendations for nurse educators came up. First of all, acceptance and positive reconsideration of occupational stress are important coping techniques for nurse educators’ mental health. Good stress management improves a person’s self-esteem and performance in a predictable world. It increased people’s motivation and self-confidence by giving them something to look forward to (Ganapathi et al. 2023).

Nurse educators are supposed to be able to encourage and motivate one another to establish a productive atmosphere that may decrease nurses’ stress (Mutianingsih et al. 2024). Another method is to encourage a productive working culture, which can promote organisational support for increased workability (Shen & Slater 2021). Both managers and staff can ensure the implementation of this approach. Rinne et al. (2024) found that the self-help intervention for educators enhances the occupational well-being of nurse educators through adaptable well-being activities suitable for diverse environments, including remote settings, without requiring ongoing support. Early career educators benefit most, though addressing workload challenges is crucial for improved usability and effectiveness.

## Discussion

Globally, there has been limited research on the factors that contribute to a lack of WLB among nurse educators. Work-related characteristics play a significant role in shaping perceptions of WLB (Sarwar et al. 2021). Various factors, such as occupational stress, contribute to an imbalance between work and personal life which can negatively impact their overall well-being. Nurse educators struggle more than other professionals to strike a healthy balance between their work and family life due to high workloads, and stress, which can result in burnout (Laari et al. 2024). Mutianingsih et al. (2024) describe stress as psychological or physical tension that can be caused by physical, emotional, social, economic or work conditions, events or experiences that are difficult to manage. The authors further allude that occupational stress is an unfavourable psychological condition as a response to pressure from the work situation. This has significant implications for nurse educators and directly affects student learning outcomes.

Due to the high-stress levels often associated with the teaching profession, nurse educators may find it difficult to adequately balance their professional responsibilities with their social and domestic commitments (Jimenez & Viloria 2024). There are several factors contributing to these difficulties, including teaching, curriculum design, administrative tasks and research. The amount of work required has increased, as has the number of hours worked. Nurse educators continue to struggle with finding a balance between an employee’s professional and personal life. Although the term ‘work–family balance’ is often used in organisations, nurse educators have focused less on it compared to segmented and guided work–family connection mechanisms (Sarwar et al. 2021). As a valued resource, nurse educators are instrumental in institutional procedures and achieving key objectives, making them a backbone in the organisation’s success. According to Fuller and Mott-Smith (2017), a notable disconnect exists between the perspectives of nurse educators and students regarding obstacles to student success, with nurse educators citing competency issues as the primary barrier.

Erasmus et al. (2024) highlight that achieving WLB presents significant challenges for nurse educators, who often view their difficulties as personal rather than social ones. While pursuing WLB is objective, employers in academic settings need to improve workplace policies. This includes offering paid maternity leave, affordable and high-quality childcare and provide social support. Furthermore, it is recommended that supervisors of nurse educators show compassion and support when addressing challenges that affect their staff.

Work–family balance, job demands, psychological capital, and job resources have been identified as crucial factors through importance-performance analysis (Kotcherlakota et al. 2017; Sarwar et al. 2021). The authors further indicate that less experienced educators are more likely to be motivated and exhibit positive attitudes toward adopting and utilising modern technology. The three most highlighted concerns in this context are technology-based communication, collaboration and time management (Kotcherlakota et al. 2017). These findings raise questions about the future feasibility of remote work, particularly as less experienced educators tend to demonstrate greater motivation and openness to adopting new technologies. Hayes et al. (2021) linked remote work during the pandemic to increased stress and burnout, emphasizing the need for organizational support.

A study done by Singh et al. (2020) showed that WLB, workload, resources and flexibility are some of the reasons why nursing educators face occupational stress, including burnout. Research on seasoned academic nurses in the later stages of their careers is limited, with most studies concentrating on nurses transitioning into academic roles. Laari et al. (2024) concurred that the results of the study revealed light on the factors that influence new nurse educators, workload and stress levels. New nurse educator’s workload and stress levels are influenced by several factors, including teaching multiple courses, large class sizes and courses, having large class sizes long hours. Urbina-Garcia (2020) highlighted that there is strong evidence that the pressures placed on the higher education industry globally today are having a detrimental effect on the mental health of academics nationwide. Employee mental illness is a result of the actual academic load.

Psychological and physical effects on academics have the potential to significantly lower productivity. Agha (2017) found that work–life conflict is significantly linked to decreased job satisfaction, whereas fostering a better WLB is strongly correlated with increased job satisfaction and overall improvement in both work and personal spheres. As a result, the findings of this study support those of earlier investigations. Then, WLB programmes should be implemented by organisations to integrate and balance work and personal life. Shen and Slater (2021) discovered that academics’ low emotional well-being and average mental health were greatly impacted by their occupation of work. Coping strategies like acceptance and positive reframing have a significant effect on academics’ mental health.

## Recommendations

Several studies have been conducted on WLB, but few of them discuss the dearth of WLB among nurse educators. The fact that most of the research was conducted internationally suggests that there is still a WLB gap in both the Republic of South Africa and sub-Saharan Africa. These topics ought to be investigated further at the national level to gain a deeper understanding of WLB.

### Limitations

The review’s reliance on six databases may have introduced selection bias, limiting the scope of the findings and constraining the argument about the selected studies’ contributions to addressing the factors affecting WLB among nurse educators. Furthermore, the scoping review lacks an in-depth analysis of the research included may have further restricted the breadth of its conclusions.

## Conclusion

The purpose of the scoping review was to explore the factors affecting WLB among nurse educators. The scoping review’s conclusions identified the factors affecting WLB among nurse educators and could assist nurse educators in dealing with factors that affect WLB. Furthermore, both the availability and utilisation of WLB programmes have been shown to reduce employee stress levels. Notably this review highlights a significant gap in existing literature and research on the factors that hinder WLB among nurse educators, with most studies focusing on clinical nurses. To address this oversight, it is recommended that future studies investigate the specific challenges faced by nurse educators, exploring the unique contributing factors that impede their work-life balance.

To encourage nurse educators to balance work and social life, strategies and programmes that focus on work-life balance must be created and put into action in a variety of settings. These strategies include effective stress management, which enhances self-esteem and performance in a stable environment. Additionally, the implementation of recommended preventative measures can help mitigate the effects of workplace stress.
